# Supplementary material for: The Predictive Role of Hepatitis B Biomarkers on HBV Reactivation following Direct-Acting Antiviral Therapy in HBV/HCV Coinfected Patients
Source: Viruses. 2022 Aug 18;14(8):1812. doi: 10.3390/v14081812 (PMC9414824; doi:10.3390/v14081812)
Supplement: Supplementary file 1 [file viruses-14-01812-s001.zip › viruses-1748586-supplementary.pdf]

Table S1. Primers for HBV RNA amplification and detection.

Round	Primer name	Sequence (5'-3')	Position
RT	HBV-3.5RNA-RT	ATTCTCAGACCGTAGCACACGACAC-CGAGATT GAGATCTTCTGCGAC	nt 2436-2415
PCR	3.5pgRNA_PCR-F	CCTACTGTTCAAGCCTCCAAGC	nt 1856-1877
	HBV RNA_PCR-R	ATTCTCAGACCGTAGCACACGACAC	random sequence
QPCR	3.5RNA_QPCR-F	AYAGACCATCAAATGCCC	nt 2295-2312
	HBV RNA_PCR-R	ATTCTCAGACCGTAGCACACGACAC	random sequence
